# Computer‐assisted beam modeling for particle therapy

**DOI:** 10.1002/mp.14647

**Published:** 2020-12-25

**Authors:** Hermann Fuchs, Alessio Elia, Andreas F. Resch, Peter Kuess, Armin Lühr, Marie Vidal, Loïc Grevillot, Dietmar Georg

**Affiliations:** ^1^ Division of Medical Radiation Physics Department of Radiation Oncology Medical University of Vienna Währinger Gürtel 18‐20 Vienna 1090 Austria; ^2^ MedAustron Ion Therapy Center Wiener Neustadt 2700 Austria; ^3^ OncoRay ‐ National Center for Radiation Research in Oncology Faculty of Medicine and University Hospital Carl Gustav Carus Technische Universität Dresden Helmholtz‐Zentrum Dresden‐Rossendorf Dresden 01309 Germany; ^4^ Helmholtz‐Zentrum Dresden‐Rossendorf Institute of Radiooncology ‐ OncoRay Dresden 01309 Germany; ^5^ Department of Medical Physics Faculty of Physics TU Dortmund University Dortmund 44227 Germany; ^6^ Center Antoine Lacassagne Nice 06189 France; ^7^ MedAustron Ion Therapy Center Wiener Neustadt 2700 Austria

**Keywords:** beam modeling, carbon ions, Monte Carlo, particle therapy, proton therapy, optimization

## Abstract

**Purpose:**

To develop a computer‐driven and thus less user‐dependent method, allowing for a simple and straightforward generation of a Monte Carlo (MC) beam model of a scanned proton and carbon ion beam delivery system.

**Methods:**

In a first step, experimental measurements were performed for proton and carbon ion energies in the available energy ranges. Data included depth dose profiles measured in water and spot sizes in air at various isocenter distances. Using an automated regularization‐based optimization process (AUTO‐BEAM), GATE/Geant4 beam models of the respective beam lines were generated. These were obtained sequentially by using least square weighting functions with and without regularization, to iteratively tune the beam parameters energy, energy spread, beam sigma, divergence, and emittance until a user‐defined agreement was reached. Based on the parameter tuning for a set of energies, a beam model was semi‐automatically generated. The resulting beam models were validated for all centers comparing to independent measurements of laterally integrated depth dose curves and spot sizes in air. For one representative center, three‐dimensional dose cubes were measured and compared to simulations. The method was applied on one research as well as four different clinical beam lines for proton and carbon ions of three different particle therapy centers using synchrotron or cyclotron accelerator systems: (a) MedAustron ion therapy center, (b) University Proton Therapy Dresden, and (c) Center Antoine Lacassagne Nice.

**Results:**

Particle beam ranges in the MC beam models agreed on average within 0.2 mm compared to measurements for all energies and beam lines. Spot sizes in air (full‐width at half maximum) at all positions differed by less than 0.4% from the measurements. Dose calculation with the beam model for the clinical beam line at MedAustron agreed better than 1.7% in absolute dose for a representative clinical case treated with protons. For protons, beam model generation, including geometry creation, data conversion, and validation, was possible within three working days. The number of iterations required for the optimization process to converge, was found to be similar for all beam line geometries and particle types.

**Conclusion:**

The presented method was demonstrated to work independently of the beam optics behavior of the different beam lines, particle types, and geometries. Furthermore, it is suitable for non‐expert users and requires only limited user interaction. Beam model validation for different beam lines based on different beam delivery systems, showed good agreement.

## 1 INTRODUCTION

Monte Carlo (MC) particle transport simulations are a useful tool in radiation oncology. In clinical treatment planning systems (TPS), a shift from semi‐analytical dose calculation approaches toward MC‐based dose calculation is ongoing, propelled by the higher achievable accuracy. Furthermore, MC simulations of a beam line help to provide additional parameters, reduce measurement time and consequently speed up the commissioning process.^1^ Especially in research, MC simulations provide unique opportunities to investigate systems where measurements would be challenging or infeasible.

Potential application are, for example, determining the dose, linear energy transfer, or secondary particle distribution inside a patient or detector system, or to test novel treatment or detector concepts. For carbon ions, the calculation of energy spectra is required for the computation of relative biological effectiveness. So far, MC simulations are the method of choice for this task.

Accurate MC simulations require the characterization of beam parameters such as spot size, beam energy and energy distribution along the whole clinical relevant beam path. Typical required measurements include laterally integrated depth dose curves (IDC) in water to determine the initial beam energy, as well as lateral beam profiles in air at various positions at and in the vicinity of the isocenter to characterize beam spreading.^1^, ^2^, ^3^ During commissioning of a light ion beam therapy (LIBT) facility, these beam parameters and the performance of a system are measured, constituting the baseline data for beam line modeling.^4^, ^5^, ^6^

Newly built LIBT centers are based on the spot scanning approach.^7^, ^8^ Although implementation details vary, the basic concept is similar: a single treatment pencil beam is scanned over the tumor volume, in layers of constant energy. A typical treatment plan consists of several energies and lateral spot positions.

A single treatment beam, that is, a pencil beam, is a set of particles, which is characterized by its position and momentum distribution. The initial characteristics of a pencil beam are defined by the accelerator, geometry of the beam line, and settings of the focusing and deflection magnets. In accelerator physics, this can be described using the Courant–Snyder strong focusing formula.^9^ It assumes undisturbed beam transport in vacuum. However, the presence of air in the irradiation room introduces additional scattering. Furthermore, additional scattering occurs in beam line elements, for example, dose monitor chambers, vacuum windows, passive beam modifying elements, all increasing the deviations from the vacuum approach before the beam impinges on the patient. Consequently, corrections are required. Next after leaving the vacuum tube, the beam evolves by passing through scatters, for example, material from beam monitoring devices or air, until impinging on the patient. This may lead to a complex overall beam shape, which requires corrections. Fermi–Eyges theory^10^ can describe such a beam propagation.

In general, beam modeling can be subdivided into four major parts: First, the geometry of the beam line is generated, including detailed models of all devices within the beam line. A full geometry model allows in addition for simulating secondary particles created in the nozzle, such as neutrons and electrons, and influences by the beam passing through the beam line, such as single elastic scattering of primary particles. As this involves the use of — often proprietary — design documents, this step is sometimes omitted starting simulations after nozzle exit. However, this limits the informative value as, for example, secondary particles produced in the nozzle, are not taken into account. Second, the mean energy and energy spread of the treatment beam are matched with measured data, for example, by comparison with integrated depth doses. Third, the parameters of the beam optics, resulting, for example, in the spot sizes at various positions and energies, within the beam line are modeled. Lastly, a calibration in terms of monitor units is performed.

So far, the modeling of a specific beam line is a tedious and time‐consuming work, but defines and limits the potential accuracy of any calculation or simulation, respectively. In commercial treatment planning systems, beam modeling is usually performed by the vendor, after providing the required measurement datasets for characterization. For self‐developed systems, for example, for quality assurance purposes, independent dose calculation or in most research systems beam modeling is left to the user. The same holds true for general purpose MC codes such as Geant4 or Fluka, or specialized implementations or interfaces such as VNCPro, MCsquare, TOPAS, or GATE.^11^, ^12^, ^13^, ^14^, ^15^ Multiple beam modeling approaches specialized to specific systems were discussed in literature,^1^, ^3^, ^16^, ^17^, ^18^, ^19^, ^20^, ^21^ which differ in the required beam model parameters and procedures. Consequently, manually tuning of beam model parameters to create a suitable agreement is possible, but a time‐consuming task requiring a lot of expertise.^3^

The possibility to create new beam models in a short time frame is especially useful in research and for sharing beam models. For instance, a beam model including the treatment nozzle may contain detailed proprietary information of the manufacturer and, consequently, cannot be easily shared. Employing a mostly automated, easy to use beam modeling method, an equivalent beam line model can be generated, having the same energy and optics behaviors, without containing proprietary data. Further potential applications are the investigation of the impact of nozzle elements on secondary particle spectra.

In this study, a method (AUTO‐BEAM) is presented to semi‐automatically determine beam model parameters for active scanning systems using the MC tool‐kit GATE, based upon Geant4. The generic AUTO‐BEAM approach can be applied to different existing beam lines and accelerators, that is, synchrotron‐ as well as cyclotron‐based facilities. It allows for the consideration of extensive nozzle geometries such as the vacuum window, ionization chambers, or ripple filters. However, the method was also tested for simpler configurations, for example, to model the beam after the nozzle exit.

AUTO‐BEAM was applied and tested for proton and carbon ion beam lines. In total, five different research as well as clinical beam lines for proton and carbon ions of three different light ion beam and proton beam therapy centers using synchrotron or cyclotron accelerator systems were modeled: (a) MedAustron ion therapy center, (b) University Proton Therapy Dresden, and (c) Center Antoine Lacassagne Nice. In addition, the performance of the beam model was benchmarked against measurements for a clinical beam line by recalculating a clinical treatment plan.

## 2 MATERIALS AND METHODS

After a short introduction into beam modeling (Section 2.A), the AUTO‐BEAM method to semi‐automatically generate beam model parameters is presented (Sections 2.B and 2.C). Second, different beam lines are modeled and evaluated (Section 2.D).

### 2.A Introduction into beam modeling

The following provides a brief overview of the basics of beam transport and modeling, for more see specific literature.^9^, ^10^, ^21^, ^22^

The energy distribution of a treatment beam can be quantified by a Gaussian function, with a mean energy and energy spread. A commonly used approach to determine the beam energy and energy spread is the measurement of IDCs in water. The energy of the ion beam directly defines its range, while the width of the Bragg peak, relates to the energy straggling in material, as well as to the energy distribution of the beam.^1^

**Fig. 1:**
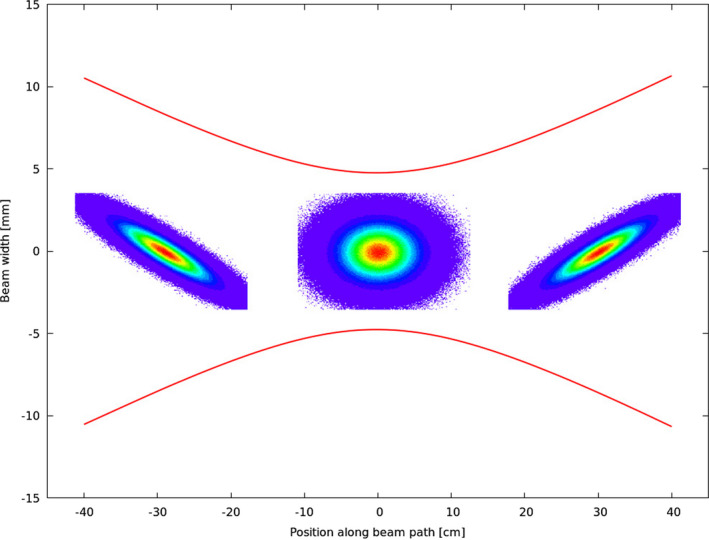
Illustration of beam size evolution (red lines) depending on the rotation of the phase space ellipse (inlay pictures in the center, depicting x over $x^{\prime }$), based on MC simulations for a low‐energy proton beam with exaggerated beam optics parameters.

The initial shape and characteristics of a pencil beam are influenced by the accelerator and beam transport system. In a phase space diagram, an ellipse can be drawn that encompasses the momentum and position distributions of the beam particles. The area of this ellipse is the beam emittance (see Fig. 1). Besides beam convergence, three parameters are required to describe all possible orientations of such an ellipse: beam width, divergence, and emittance. The beam waist is defined as the point where a converging beam has the smallest diameter before it starts to diverge. There, the shape of the encompassing phase space ellipse is upright, that is, the major axes of the ellipse are orthogonal to the coordinate system axes.

Only in this case, the emittance can be calculated as follows [Eq. (1)]:

(1) ϵ=σ·θ·π

where *ε* is the beam emittance in (mm mrad), *σ* the sigma of the Gaussian beam width distribution in (mm), and *θ* the beam divergence in (mrad). At any other point, the beam ellipse appears tilted, more precisely, sheared, with the emittance containing information on the ellipse rotation, that is, on the particle position and momentum distribution. In an undisturbed system (e.g., no scattering), the area of the beam ellipse in phase space is conserved, while beam width and divergence change accordingly during propagation.

### 2.B Monte Carlo simulations

To calculate the beam propagation in air and water, MC simulations were employed using GATE‐RTion v1.0 alongside GEANT4 v10.3p03. GATE‐RTion is a project of the OpenGATE collaboration aiming to provide a validated, stable, and long‐term release of the MC tool‐kit GATE, intended for dosimetric applications in LIBT facilities. GATE‐RTion v1.0 is based on GATE v8.1, which is a free and open‐source general‐purpose MC tool kit, developed by the OpenGATE collaboration since 2001.^15^, ^23^ It allows for simulating full radiation therapy treatment plans using a respective beam model.^21^, ^24^ MC parameters used for simulations in this manuscript are listed in Table I.

**Table I:** Parameters employed for Monte Carlo simulation and optimization.

Parameter		Value
Physics list	protons	QGSP_BIC_EMZ
	carbon ions	Shielding_EMZ
Production cuts	air	10 m
	material	0.1 mm
Number of primary particles	IDC	3e5(p) 2.5e5(C12)
	beam width	2e6(p) 5e5(C12)
Optimization stop criterion	parameter change	<1e‐4
	weighting function value	<1e‐5
Bin size	integrated depth dose curve	0.1 mm
	beam width	0.5 ${\text{mm}}^{2}$

In this study, two specialized source models for ion therapy offered by GATE were employed, namely the pencil beam source (PBS), and the treatment planniny system pencil beam source (TPS‐PBS). The PBS is intended to define beam parameters for a single beam. Parameters include the particle type, initial energy, energy distribution, spot size, divergence, and emittance per incident plane. For more complex treatment fields, the TPS‐PBS is used. It employs a polynomial parametrization of these parameters as a function of energy and allows for a set of spots with different weights, positions, and energies.^21^

### 2.C Beam model optimization — AUTO‐BEAM

AUTO‐BEAM consists of the following sequential stages: setup of calculation geometry, energy tuning, beam optics tuning, and dose calibration. A complete set of beam model parameters was determined including mean energy, energy spread, as well as for both optical planes *σ*, *θ*, and *ε*. The parameter tuning process iteratively determined optimal beam model parameters starting with an initial guess of parameters. The agreement between measured and simulated data resulting from the current parameter set was evaluated by a weighting function. Based on this evaluation, a new set of starting parameters was generated and a new simulation was performed until a user defined agreement threshold was reached. Tuning was performed for a selected number of nominal beam energies. Based on these data, a beam model was generated for the whole clinical range.

The computer‐driven beam modeling framework was implemented in Python based on the pygmo framework in version 2.7^25^ avoiding any commercial software packages. It features a large selection of gradient‐based and evolutionary optimization algorithms. A Nelder–Mead simplex optimization algorithm (pygmo:nlopt:neldermead)^26^ was used. Given the stochastic nature of MC simulations, this derivative‐free algorithm helps to avoid unpredictable behavior. However, it is susceptible to converge to local minima, potentially introduced by the variation within the MC simulation results. Consequently, an evolutionary‐based improved harmony search algorithm (pygmo:ihs, 500 generations considered, cross over rate 0.85, mutation rate 0.35–0.99, mutation width 1e‐5‐1)^27^ was tested as an alternative in order to attempt to converge to the global minimum.

The calculation time was found to be increasing with increasing energy and the complexity of the geometries within the beam path. MC parameters (see Table I) were chosen to allow a single MC simulation (e.g., one iteration) for protons in about 2 min, averaged over the whole energy range. For carbon ions, simulation time to achieve a comparable quality was considerably longer, resulting in overall longer optimization times. Calculations were performed using an Intel i7‐6700K, 4.0 GHz.

#### 2.C.1 Geometry creation

In a first step, the geometry of the beam line was created in GATE. It contained a virtual beam starting point (source position), as well as materials alongside the beam path. The modeling of the beam line either included a modeled nozzle or started directly after the beam exit window, depending on the requirements of the user and available geometry information. For optics tuning, the beam was passing through air. The lateral profile positions in the simulations aligned to the corresponding measurement positions. For energy tuning, the beam impinged on a water phantom with the phantom surface located at isocenter. This protocol is similar to base line data measurements for treatment planning system commissioning. Simulations were designed to create output at the same positions and in similar data format compared to the experimental data.

Evaluation of data was performed by dedicated software developed in Python or C++ employing the ROOT framework.^28^, ^29^ The same evaluation tools were used to analyze measured and simulated data.

#### 2.C.2 Beam energy tuning

Modeling of the beam energy was performed first and used as a basis for the beam optics modeling due to the energy dependence of scattering. A Gaussian function was employed to model the energy distribution of the beam. Energy (*E*) and energy spread ($E_{\sigma }$) are two independent quantities, potentially allowing a simultaneous optimization. However, the measurement data employed for characterization using range or Bragg peak width are weakly correlated, as, for example, a larger particle range leads to more range straggling, consequently also increasing the Bragg peak width. Therefore, it was found to be more efficient to perform the modeling sequentially.

For range and peak assessment, laterally integrated depth dose curves (IDC) in water were used. The beam range, defined at 80% of the intensity maximum beyond the peak ($R_{80}$) as well as the peak width at 50% (${\text{BPW}}_{50}$) and 80% (${\text{BPW}}_{80}$) of the peak maximum were determined using linear interpolation. For optimization of beam energy and energy distribution, the $R_{80}$ and the ${\text{BPW}}_{80}$ parameter were considered, respectively. The ${\text{BPW}}_{50}$ was only used for evaluation.

For beam energy tuning, the least square based weighting function

(2) $$f\left(E\right)={\left[R_{80,\mathrm{meas}}^{E}‐R_{80,\mathrm{sim}}\left(E\right)\right]}^{2}$$

was minimized, where $R_{80,\mathrm{meas}}^{E}$ and $R_{80,\mathrm{sim}}\left(E\right)$ are the respective measured and simulated measurement values for $R_{80}$ of energy *E*.

For energy distribution tuning, employing the same approach, the function

(3) $$f^{E}\left(\sigma_{E}\right)={\left[BPW_{80,\mathrm{meas}}^{E}‐BPW_{80,\mathrm{sim}}^{E}\left(\sigma_{E}\right)\right]}^{2}$$

was minimized, where $BPW_{80,\mathrm{meas}}^{E}\left(\sigma_{E}\right)$ and $BPW_{80,\mathrm{sim}}^{E}\left(\sigma_{E}\right)$ are the measured and simulated measurement values for ${\text{BPW}}_{80}$ for energy *E*, respectively using the energy distribution $\sigma_{E}$.

#### 2.C.3 Beam optics tuning

With three input parameters (*σ*, *θ*, and *ε*), beam optics tuning is a multidimensional optimization problem, making it increasingly susceptible to small fluctuations caused by the MC simulations. The lateral beam profiles in air were obtained at different distances from the isocenter using so called one‐dimensional dose actors. To determine the beam size, the full‐width‐at‐half‐maximum (FWHM) of lateral beam profiles was evaluated in the horizontal and the vertical plane.

A similar iterative approach as for beam energy tuning was employed, but two weighting functions were tested. The first one used a least square based weighting function *f*(*E*) similar as applied for beam energy tuning,

(4) $$f_{E}\left(\sigma ,\theta ,\epsilon \right)=\sum_{i=0}^{N}{\left[FWHM_{\mathrm{meas},i}^{E}‐FWHM_{\mathrm{sim},i}^{E}\left(\sigma ,\theta ,\epsilon \right)\right]}^{2}$$

where $FWHM_{\mathrm{meas},i}^{E}$ and $FWHM_{\mathrm{sim},i}^{E}\left(\sigma ,\theta ,\epsilon \right)$ are for the energy E the $i^{\mathrm{th}}$ measured and simulated FWHM values, respectively, of a total of *N* measured FWHM values at varying distances from the isocenter. In order to avoid parameter oscillation with energy, a regularization term $R^{E}\left(\sigma ,\theta ,\epsilon \right)$ was introduced, resulting in the extended weighting function $f_{r,E}\left(\sigma ,\theta ,\epsilon \right)$

(5) $$f_{r}^{E}\left(\sigma ,\theta ,\epsilon \right)=\sum_{i=0}^{N}{\left[FWHM_{\mathrm{meas},i}^{E}‐FWHM_{\mathrm{sim},i}^{E}\left(\sigma ,\theta ,\epsilon \right)\right]}^{2}+\lambda R^{E}\left(\sigma_{i},\theta_{i},\epsilon_{i}\right)$$

where *λ* is a constant and

(6) $$R^{E}\left(\sigma ,\theta ,\epsilon \right)={\left(\sigma_{E_{j}}‐\sigma_{E_{j‐1}}\right)}^{2}+{\left(\theta_{E_{j}}‐\theta_{E_{j‐1}}\right)}^{2}+{\left(\epsilon_{E_{j}}‐\epsilon_{E_{j‐1}}\right)}^{2}$$

The subscripts ${\;}_{E_{j}}$ and ${\;}_{E_{j‐1}}$ indicate the simulation parameters of the current and the final parameters of the preceding energy, respectively. For the lowest energy, no regularization was applied. The regularization parameter, *λ*, was empirically tuned for a good compromise between flexibility of the optimization as well as reduced parameter fluctuations and was set to 0.1.

#### 2.C.4 Model generation

Based on the optimized parameters for a set of discrete energies, polynomial fits were performed for all parameters (energy, energy distribution, beam width, divergence, and emittance per incident plane), respectively, resulting in a beam model encompassing the whole energy range. Fourth‐ to sixth‐order polynomials were used to describe the energy, energy spread, and optical parameters as a function of the nominal energy.

#### 2.C.5 Dose calibration

Absolute dose calibration data were only available for the MedAustron clinical beam line. The dose delivery system at MedAustron was calibrated in the plateau region in number of particles. Further details can be found in Palmans and Vatnitsky, 2016.^4^

Absolute dose measurements were performed at 24 points located in the spread‐out Bragg peak (SOBP) of a reference three‐dimensional (3D) cubic plan of $8\times 8\times 8{\text{cm}}^{3}$, located 40 cm upstream from the isocenter (ISD 40). More specifically, doses were measured in water using the 24 Pin‐Point chamber (TM31015, PTW, Freiburg, Germany) system from PTW,^30^ repeated on 24 different days resulting in a standard deviation of less than 0.004 Gy per individual detector position. Measurements were compared to simulations using the generated beam model. A dose‐scaling factor was determined and applied to match the absolute dose in the SOBP region.

#### 2.C.6 Modeling evaluation: pilot study

The reproducibility of the optimization process was investigated by repeatedly performing the optimization for the whole dataset of the MedAustron clinical beam line using either the weighting function alone or by including regularization. In addition, the clinical impact of highly different emittance values was investigated for a 62 and a 252 MeV proton beam. Two beam models were selected with comparable agreement in beam width and divergence, while showing differences in emittance by at least a factor of two. Using both beam parametrizations, a central single proton beam was irradiated into a water phantom at isocenter. The beam widths in air and water were evaluated before and in the water phantom, respectively.

For all modeled beam lines, the obtained beam models were evaluated in terms of range ($R_{80}$), peak width (${\text{BPW}}_{50}$, ${\text{BPW}}_{80}$), as well as spot sizes in air (FWHM) at multiple distances from the isocenter for energies equally distributed within the modeled energy range. In addition, treatment plans were created with single, central spots for each of the energies of interest. Treatment plans were simulated using the GATE TPS‐PBS with the same beam source point and geometries as described above. Absolute and relative differences were considered in the comparison between measurement and simulation. The process was performed for five equally spaced energies over the available energy range.

Absolute dose agreement was tested for the clinical MedAustron beam line. Furthermore, exemplary treatment plans of a $8\times 8\times 8{\text{cm}}^{3}$ validation 3D cubic structure positioned at the isocenter (ISD 0), and a central nervous system patient case were used for model benchmarking. Treatment plans were irradiated in water. The dose was measured at multiple positions in the SOBP using the PTW 24 Pin‐Point chamber system and compared to simulations. For the validation 3D cube, additional measurements and simulations in the entrance region alongside the beam path were performed.

### 2.D Clinical beam lines

AUTO‐BEAM was employed to create beam models for four proton beam lines and one carbon ion beam line at three LIBT centers using either synchrotron or cyclotron‐based accelerators. The same evaluation tools were employed to analyze the measured and the AUTO‐BEAM generated data.

#### 2.D.1 Clinical and research beam lines at MedAustron

MedAustron, the Austrian ion‐therapy center, is a synchrotron‐based LIBT facility, featuring three patient treatment rooms (one horizontal, one horizontal and vertical, and one proton gantry treatment room) as well as a dedicated research room. All rooms use a scanned beam for 62–253 MeV protons and carbon ions (120–402 MeV/u).^31^ In addition, in the research room the clinical nozzle can be moved out of the beam path to allow higher proton beam energies up to 800 MeV. A clinical beam line was modeled including a detailed model of the clinical nozzle for proton and carbon ions.^3^ The research beam line was modeled for protons only in two configurations: first, with the source point upstream of the clinical nozzle, that is, the beam traversed the fully modeled clinical nozzle, and second, with the same source position, but with the clinical nozzle removed. This allowed to investigate a diverging and a converging beam, respectively.

The water equivalent thickness of the MedAustron clinical nozzle is 2.4 mm. The source position was selected to be upstream of the vacuum window, 130 cm before the isocenter, with the nozzle exit 65 cm before the treatment isocenter. Energy measurements were performed by IDC measurements in a water phantom positioned at isocenter.

Beam optics measurements were performed at regular isocenter distances using a scintillator screen detector with a resolution of $0.5\times 0.5{\text{mm}}^{2}$ (Lynx, IBA Dosimetry, Schwarzenbruck, Germany) and an in‐house developed evaluation software.^5^ Depending on the beam line, measurements started close to the nozzle exit (57 cm) or close to the vacuum window (118 cm, research beam line only) until 20 cm downstream of the isocenter.

For proton beam modeling, data were measured for 20 uniformly distributed energies for the clinical and research beam line. For the research beam line without nozzle, only 5 uniformly distributed energies were available. For carbon ions, data were measured for 16 uniformly distributed energies.

#### 2.D.2 University Proton Therapy Dresden, Germany

The University Proton Therapy Dresden (UPTD) operates a cyclotron‐based facility (IBA PT, Louvain‐La‐Neuve, Belgium), featuring one patient treatment room equipped with a proton gantry and a dedicated experimental room with two research beam lines. In the treatment room, a universal nozzle capable of treatment field formation in passive double‐scattering or active pencil beam scanning mode is available.

A beam model was created for the clinical nozzle in active scanning mode using a source point located directly after the nozzle exit as no blue prints of the inner layout were available. IDC measurements in a water phantom starting at the isocenter were performed to determine the beam energy and width. Beam optics measurements were performed in air using a scintillator screen detector with a resolution of $0.5\times 0.5{\text{mm}}^{2}$ (Lynx) to obtain lateral beam profiles at in total five positions per energy: at the isocenter as well as 10 and 15 cm up‐ and downstream the isocenter. Data were measured for 27 energies, spanning the energy range from 100 to 226.7 MeV.

#### 2.D.3 Center Antoine Lacassagne, France

The Center Antoine Lacassagne in Nice (CAL), France, hosts a supra‐conducting synchrocyclotron combined with a compact gantry delivering a proton scanned beam (Proteus®ONE, IBA PT, Louvain‐La‐Neuve, Belgium).

Beam energy measurements were performed by IDC measurements starting at isocenter using a large Bragg peak chamber (StingRay, IBA Dosimetry, Schwarzenbruck, Germany) in a water phantom. Beam optics measurements were performed using lateral beam profiles of monoenergetic single spot measured in air with a scintillator screen detector with a resolution of $0.5\times 0.5{\text{mm}}^{2}$ (Lynx) for 5 different depths around the treatment isocenter (0, ± 10, ± 20 cm). Spot size was analyzed by a software tool from the manufacturer that is fitting a Gaussian curve to the spot intensity. Data were measured for 26 energies, spanning the available energy range from 100 to 226 MeV.

## 3 RESULTS

Range deviations between measurement and MC simulation employing the generated beam models agreed on average within 0.2 mm, with minimum and maximum deviations within −0.3 and 0.1 mm, respectively (see Table II). For the beam line at CAL, due to a nonlinear nominal energy‐range relationship, for the lowest energies, range differences were higher, showing differences up to 0.8 mm. The modeling of the Bragg peak width showed average deviations up to 1.7% for ${\text{BPW}}_{80}$. Especially for carbon ions, ${\text{BPW}}_{50}$ deviations were higher, up to −9.1%. A comparison of range and Bragg peak width agreement can be found in Table II.

**Table II:** Absolute and relative range differences for simulation minus measurement, averaged over all available energies.

Beam line	Particle	abs. diff. $R_{80}$ avg. (mm) (min;max)	rel. diff. $R_{80}$ avg. (%) (min;max)	rel. diff. ${\text{BPW}}_{50}$ avg. (%) (min;max)	rel. diff. ${\text{BPW}}_{80}$ avg. (%) (min;max)
MedAustron research, w. nozzle	p	0.0 (−0.2; 0.1)	0.0 (0.0; 0.0)	0.8 (−3.5; 3.4)	0.6 (−2.7; 2.3)
MedAustron research, no nozzle	p	0.0 (−0.1; 0.0)	0.0 (0.0; 0.0)	1.2 (−1.2; 5.3)	−1.6 (−4.6;0.7)
MedAustron clinical	p	0.0 (−0.2; 0.1)	0.0 (0.0; 0.0)	0.8 (−3.5; 3.4)	0.6 (−2.7; 2.3)
MedAustron clinical	C12	0.0 (0.0; 0.2)	0.0 (−0.1; 0.1)	−7.1 (−9.2; −2.2)	1.7 (−1.9; 6.3)
Dresden clinical	p	−0.2 (−0.3; 0.1)	−0.1 (−0.2; −0.1)	2.2 (0.9; 4.5)	−0.5 (−2.6; 2.6)
Nice clinical	p	0.1 (−0.4; 0.8)	0.0 (−0.5; 0.3)	−3.0 (−6.0; 0.1)	−1.6 (−4.6;4.2)

Numbers in brackets denote minimal and maximal values.

**Fig. 2:**
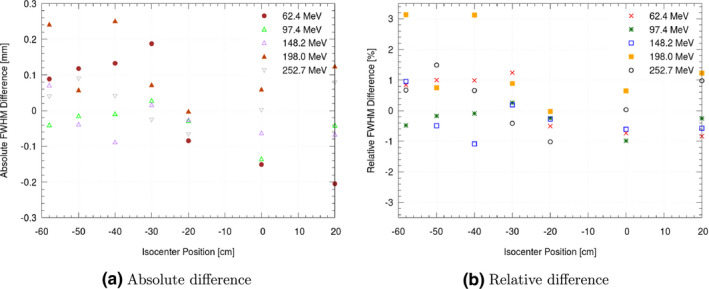
Comparison of the computer driven beam model with measured beam width showing absolute differences (a) and relative differences (b) at various positions along the beam path for five selected energies for the MedAustron clinical beam line. The beam model was generated with the source point upstream of the nozzle, i.e. simulating the full nozzle.

For all beam lines, good overall agreement in spot size was found between measurements and the data simulated with the obtained beam models (see Table III). For the MedAustron clinical beam line, a comparison of beam widths from all positions (isocentric and non‐isocentric) for five equally spaced energies showed deviations of less than 0.3 mm (Fig. 2). For all beam lines, the FWHM of the lateral beam profiles agreed on average within 1.6%, absolute FWHM difference at all positions was lower than 1.1 mm (Table III).

**Table III:** Absolute and relative difference of spot size data (FWHM) for simulation minus measurement, averaged over all measurement positions available.

Beam line	Particle	avg. abs. difference FWHM (mm) (min;max)	avg. rel. difference FWHM (%) (min;max)	RMS
MedAustron research, w. nozzle	p	0.0 (−0.3; 0.7)	−0.6 (−5.2; 2.5)	0.18
MedAustron research, no nozzle	p	0.2 (1.1; −0.5)	1.6 (−7.4;12.3)	0.13
MedAustron clinical	p	0.0 (−0.3; 0.2)	0.3 (−3.1; 1.1)	0.10
MedAustron clinical	C12	0.0 (−0.3; 0.2)	0.2 (−3.7; 3.7)	0.11
Dresden clinical	p	0.0 (−0.3; 0.2)	−0.1 (−2.0; 2.2)	0.13
Nice clinical	p	0.4 (−0.1; 0.4)	0.6 (−1.2; 4.8)	0.15

Calculated over 5 regularly spaced energies over the available beam energy range. Numbers in brackets denote minimal and maximal values.

The number of iterations required to achieve convergence was found to depend weakly on the beam line geometry but was independent of the employed particle type. On average employing the Nelder–Mead simplex algorithm, for all modeled beam lines 30, 22, and 108 iteration steps were required for energy, energy spread, and optics (one plane) optimization, respectively. Using the improved harmony search algorithm required more than a factor of 100 additional iterations. Total beam modeling for a beam line, including setup, data conversion, model generation, and evaluation, could be performed in less than 3 days, for protons. For carbon ions, the used simulation settings were not optimized for speed, resulting in about 5 days.

**Fig. 3:**
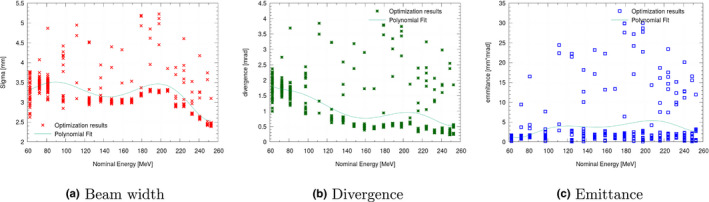
Results of beam width (a) beam divergence (b) and beam emittance (c) for multiple optimizations for a clinical beam line at MedAustron. Note the amount of variance introduced due to the combination of weakly correlating emittance and simulation uncertainties.

**Fig. 4:**
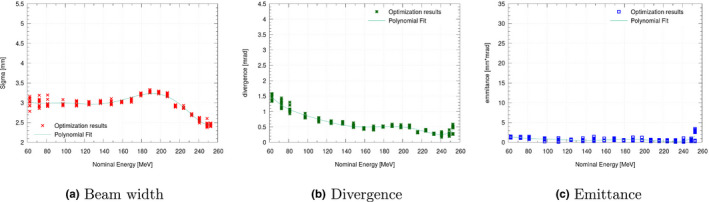
Results of beam width (a) beam divergence (b) and beam emittance (c) for multiple optimizations for the clinical beam line at MedAustron using regularization.

**Fig. 5:**
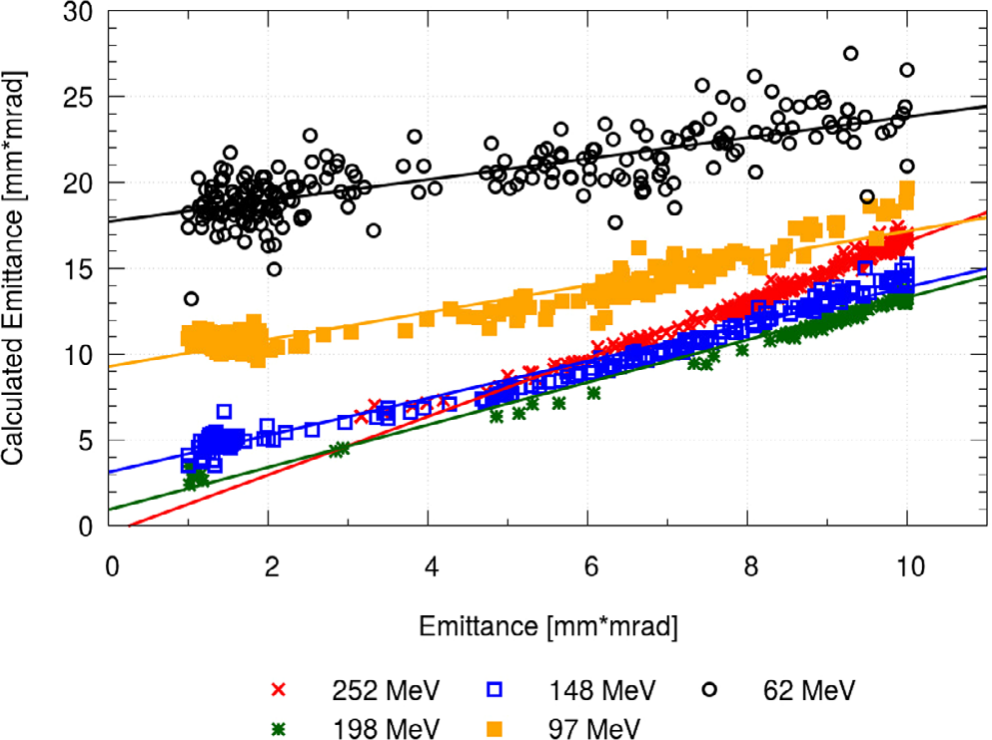
Multiple optimization results for the research beam line at MedAustron without regularization. Every dot represents the emittance calculated using Eq. (1) plotted over the emittance resulting from multiple optimization runs.

Repeated optimization runs on the same datasets revealed a noticeable variation of the weighting function (see Fig. 3), which decreased substantially when applying regularization (see Fig. 4). On average, a reduction in standard deviation of the respective parameters by a factor of 7.8–10.2 for sigma, divergence, and emittance was possible due to regularization. A fit through both sets of optimization results was performed (see in Figs. 3 and 4), resulting in beam models, with comparable good agreement over the whole clinical energy range. Due to the increased variance, beam model generation without regularization was found to be computational more intensive as additional repetitions were required. A potential correlation between the emittance values obtained by AUTO‐BEAM and the emittance as calculated by Eq. (1) is shown in Fig. 5.

**Fig. 6:**
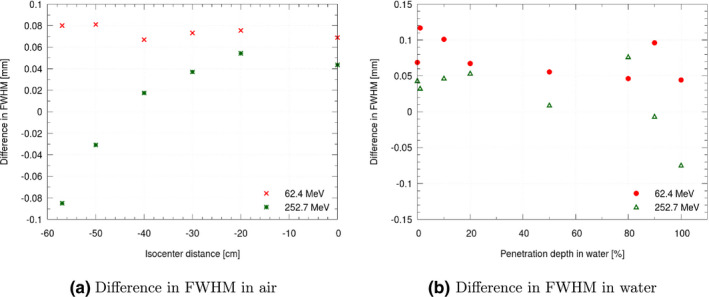
Difference between two beam models with a similar beam width differences in air (a) and water (b) using emittance values which are differing by a factor of two, for two exemplary proton energies.

A comparison of two beam models with a similar beam width behavior in air but with emittance values differing by a factor of two can be seen in Fig. 6. The observed differences in air as well as in water close to the Bragg peak are negligible.

In the SOBP region of the reference 3D cubic plan, an average absolute dose difference of −3.5% was found and subsequently used as a dose rescaling factor for the other simulated plans. After applying this correction, for the validation 3D cube at ISD 0, measurement points over the whole beam path agreed on average within 0.5%. The agreement was less good for the clinical CNS case, with average deviations within 1.72% compared to the measured values. Detailed results of the 3‐dimensional verification can be found in Table IV.

**Table IV:** Relative dose difference of simulation minus measurement for the clinical MedAustron beam line.

Plan name	Description	avg. local difference (%) (min;max)	RMS
validation 3D cube (ISD 0)	$8\times 8\times 8{\text{cm}}^{3}$	0.48 (−1.88;2.68)	0.009
CNS patient	field 1	1.72 (−0.25;6.16)	0.024
CNS patient	field 2	1.25 (−4.72;4.78)	0.022

Doses were measured in water at different points of interest using a 24 Pin‐Point chamber system.

## 4 DISCUSSION

The presented computer‐driven AUTO‐BEAM method allows straightforward generation of beam models. It was shown to work for different beam line and accelerator designs. AUTO‐BEAM is based on an iterative optimization process. It does not take into account more specialized methods to create beam optics modeling as described in literature.^16^, ^21^ As mentioned above, the Courant–Snyder strong focusing formula^9^ would be an efficient method for parametrization of the measurement data to derive beam optics parameters. However, the scattering occurring due to the presence of air or other elements in the beam path (dose monitor chambers, vacuum windows) requires complex corrections. Deviations in spot size due to the presence of air depend on the beam line and were found to be up to 2.5 mm for some of the modeled beam lines, which exceeds the clinical requirements.

The presented AUTO‐BEAM method takes all these effects into account by using MC simulations for beam propagation. Consequently, it is slower and computationally more expensive for geometries where specialized solutions can be employed. At the same time, AUTO‐BEAM is a general approach and therefor, not restricted to a single application. It allows to account for complex beam modeling devices, such as ripple filters, alongside with detailed models of a clinical nozzle.

Two beam models were optimized for the research beam line at MedAustron starting at nozzle exit or nozzle entrance. Beam models of similar quality were achieved in terms of the measured energy and beam optics parameters. It was possible to accurately model the beam core (Gaussian‐shaped center) of the beam with and without tracking of particles through the nozzle. The authors want to underline that AUTO‐BEAM aims only at generating beam models. The influence on the beam halo or change of particle spectrum were not subject of this study.^32^

The polynomial description of beam parameters is required by the GATE TPS source. In current practice, beam optics parameters are fine‐tuned only for a subset of energies by the accelerator physicists. Parameters for other energies are interpolated, a practice similarly done at other accelerators. This can lead to a complex function behavior, potentially requiring high order polynomials for an accurate description (see Fig. 4). In this manuscript, sixth‐order polynomials were chosen as upper limit to avoid over‐fitting, and were found to be sufficient. Overall, the range‐energy relationship could be modeled easily. For a single beam line (CAL), a complex nominal energy to range relationship for the lowest energies was difficult to describe using only a sixth‐order polynomial. Although a higher order polynomial may have solved this issue, we decided to limit the model to avoid over‐fitting. Consequently, energy range deviations up to 0.8 mm were found and accepted for this beam line. ${\text{BPW}}_{50}$ was found to show higher deviations. However, this was not related to the initial energy spread, but may originate from inherent limits on physical models used for MC simulations, leading to slightly different Bragg peak shapes and was especially pronounced for carbon ions.

Absolute dose difference was evaluated in the SOBP region and a scaling factor was applied. This absolute dose offset between measurement and simulation arises from minute differences in the underlying Geant4 physics processes and dose calibration uncertainties. Cubic plans were measured repeatedly on different days, while the clinical treatment plans were measured only once.

The software architecture was designed to be flexible, also allowing for exchanging the GATE MC tool kit with other calculation methods. Despite its advantages, using a MC code has drawbacks, such as relatively long calculation times and fluctuations due to the statistical nature of the method. This causes problems for the beam model optimization process, as from a mathematical point of view the optimization function is not continuous. Increasing the number of particles simulated is an obvious solution to the problem, but increases the calculation time. Depending on simulation uncertainty, gradient‐based and many gradient‐free methods may not converge or converge to different points due to non‐continuous effects for small parameter changes. In our case, the number of particles per iteration (see Table I) was chosen rather low, to reduce calculation time. The inherent numerical instability of MC simulations and the observed sensitivity of the beam model to the absolute emittance, resulted in a considerable variance. Small changes in the initial beam width or divergence parameters were to some extent compensated by large changes in emittance. This could also be seen for the two selected beam parametrizations with widely differing emittance values (see Fig. 6). The reduction of variance by introducing regularization allowed for fewer repetitions while ensuring a similar beam model quality.

Energy optimization, required no regularization, as it was implemented using quasi‐independent parameters (mean energy and energy spread), creating one‐dimensional problems of energy tuning. In principle, using regularization a single optimization run should be sufficient to generate an accurate model. However, especially for new geometries and physics settings influencing the variance, we recommend to use at least three optimization runs to be able to judge on the repeatability. Non‐gradient based algorithms, that are specifically designed for such integer‐class problems were tested for the optimization, but they required substantially more (more than a factor of 100) iterations resulting in considerably longer optimization times, making this approach practically unfeasible.

In literature, sometimes only two beam optics parameters are used, namely, beam width and divergence.^12^, ^22^, ^33^ Consequently, an erect beam parameter ellipse with the relationship between beam width and divergence [see Eq. (1)] is sufficient at the beam waist position [see Eq. (1)]. The waist position is then chosen such that the beam properties match the measurements at a given point (ICRU Report 35^22^). However, this approach is limited, when additional positions along the beam path are taken into account, or the underlying assumption of a diverging beam is not valid. In a converging beam, as reported by clinical treatment systems for protons,^16^ the waist position obviously cannot be chosen arbitrarily.

Under clinical conditions, the necessity of specifying the emittance is weakened, as scattering which occurs during propagation in the nozzle and air, reduces this effect. Consequently, the absolute value of the emittance appeared to be less important than the relation between beam width and divergence, providing more flexibility in determination of the beam emittance. An energy‐dependent correlation between modeled and calculated emittance was found, which might be interpreted as the tilt of the beam phase space ellipse (see Fig. 5).

The successful generation of beam models for different beam lines, beam geometries, as well as for two particle species, showed the potential and validity of AUTO‐BEAM. For the clinical MedAustron proton beam line, a manual beam model was created before.^3^ Its agreement in the isocenter is close to the model generated in this manuscript. However, it required considerable manpower and knowledge for generation. At other positions not considered in the manual modeling, such as close or far away from the nozzle, small deviations can be found. It seems clear that while a beam model of similar quality can be generated manually; however, the patience and endurance of an algorithm cannot be easily matched.

## 5 CONCLUSION

The presented AUTO‐BEAM method allowed for creating full Monte Carlo based beam models with limited manual interaction. It was tested using experimental data of one carbon ion and four different proton beam lines at three different institutions using synchrotron or cyclotron‐based accelerators. The method was shown to work independently of an existing nozzle model or beam optics design. The observed agreement of the resulting beam models is promising for clinical as well as research purposes.

## CONFLICTS OF INTEREST

The authors have no conflicts to disclose.

## References

[1] Parodi K , Mairani A , Brons S , et al. Monte Carlo simulations to support start‐up and treatment planning of scanned proton and carbon ion therapy at a synchrotron‐based facility. Phys Med Biol. 2012;57:3759‐3784. 22617050 10.1088/0031-9155/57/12/3759

[2] Clasie B , Depauw N , Fransen M , et al. Golden beam data for proton pencil‐beam scanning. Phys Med Biol. 2012;57:1147. 22330090 10.1088/0031-9155/57/5/1147 PMC3387676

[3] Elia A . Characterization of the GATE Monte Carlo platform for non‐isocentric treatments and patient specific treatment plan verification at MedAustron ‐ Vienna ‐ Austria. Ph.D. thesis; 2019.

[4] Palmans H , Vatnitsky SM. Beam monitor calibration in scanned light‐ion beams. Med Phys. 2016;43:5835‐5847. 27806608 10.1118/1.4963808

[5] Grevillot L , Stock M , Palmans H , et al. Implementation of dosimetry equipment and phantoms at the MedAustron light ion beam therapy facility. Med Phys. 2018;45:352‐369. 29105791 10.1002/mp.12653

[6] Mirandola A , Molinelli S , Freixas VG , et al. Dosimetric commissioning and quality assurance of scanned ion beams at the Italian National Center for Oncological Hadrontherapy. Med Phys. 2015;42:5287‐5300. 26328978 10.1118/1.4928397

[7] Lomax AJ , Böhringer T , Bolsi A , et al. Treatment planning and verification of proton therapy using spot scanning: Initial experiences. Med Phys. 2004;31:3150‐3157. 15587667 10.1118/1.1779371

[8] Pötter R , Balosso J , Baumann M , et al. Union of light ion therapy centers in Europe (ULICE EC FP7) ‐ Objectives and achievements of joint research activities. Radiother Oncol. 2018;128:83‐100. 30001932 10.1016/j.radonc.2018.04.027

[9] Courant E , Snyder H. Theory of the alternating‐gradient synchrotron. Ann Phys. 2000;281:360‐408.

[10] Gottschalk B . Techniques of proton radiotherapy: transport theory. Tech Rep. 2012. arXiv:1204.4470.

[11] Souris K , Lee JA , Sterpin E. Fast multipurpose Monte Carlo simulation for proton therapy using multi‐ and many‐core CPU architectures. Med Phys. 2016;43:1700‐1712. 27036568 10.1118/1.4943377

[12] Battistoni G , Bauer J , Boehlen TT , et al. The FLUKA code: an accurate simulation tool for particle therapy. Front Oncol. 2016;6:4273‐4289. 10.1088/0031-9155/55/15/006. PMC4863153 27242956

[13] Perl J , Shin J , Schümann J , Faddegon B , Paganetti H. TOPAS: An innovative proton Monte Carlo platform for research and clinical applications. Med Phys. 2012;39:6818‐6837. 23127075 10.1118/1.4758060 PMC3493036

[14] Fippel M , Soukup M. A Monte Carlo dose calculation algorithm for proton therapy. Med Phys. 2004;31:2263‐2273. 15377093 10.1118/1.1769631

[15] Jan S , Benoit D , Becheva E , et al. GATE V6: a major enhancement of the GATE simulation platform enabling modelling of CT and radiotherapy. Phys Med Biol. 2011;56:881‐901. 21248393 10.1088/0031-9155/56/4/001

[16] Almhagen E , Boersma D , Nyström H , Ahnesjö A. A beam model for focused proton pencil beams. Phys Med 2018:52,27‐32. 10.1016/j.ejmp.2018.06.007 30139606

[17] Grassberger C , Lomax A , Paganetti H. Characterizing a proton beam scanning system for Monte Carlo dose calculation in patients. Phys Med Biol. 2015;60:33‐645. 10.1088/0031-9155/60/2/633 PMC4300341 25549079

[18] Parodi K , Mairani A , Brons S , et al. The influence of lateral beam profile modifications in scanned proton and carbon ion therapy: a Monte Carlo study. Phys Med Biol. 2010;55:5169‐5187. 20714044 10.1088/0031-9155/55/17/018

[19] Stankovskiy A , Kerhoas‐Cavata S , Ferrand R , Nauraye C , Demarzi L. Monte Carlo modelling of the treatment line of the Proton Therapy Center in Orsay. Phys Med Biol. 2009;54:2377‐2394. 19321923 10.1088/0031-9155/54/8/008

[20] Paganetti H , Jiang H , Parodi K , Slopsema R , Engelsman M. Clinical implementation of full Monte Carlo dose calculation in proton beam therapy. Phys Med Biol. 2008;53:4825‐4853. 18701772 10.1088/0031-9155/53/17/023

[21] Grevillot L , Bertrand D , Dessy F , Freud N , Sarrut D. A Monte Carlo pencil beam scanning model for proton treatment plan simulation using GATE/GEANT4. Phys Med Biol. 2011;56:5203‐5219. 21791731 10.1088/0031-9155/56/16/008

[22] Ibbott GS. Radiation dosimetry: electron beams with energies between 1 and 50 MeV (ICRU Report No. 35). Med Phys. 1985;12:813.

[23] Sarrut D , Bardiés M , Boussion N , et al. A review of the use and potential of the GATE Monte Carlo simulation code for radiation therapy and dosimetry applications. Med Phys. 2014;41:064301. 24877844 10.1118/1.4871617

[24] Grevillot L , Bertrand D , Dessy F , Freud N , Sarrut D. GATE as a GEANT4‐based Monte Carlo platform for the evaluation of proton pencil beam scanning treatment plans. Phys Med Biol. 2012;57:4223‐4244. 22684098 10.1088/0031-9155/57/13/4223

[25] Pygmo scientific library. https://esa.github.io/pagmo2.

[26] Nelder JA , Mead R. A simplex method for function minimization. Comput J. 1965;7:308‐313.

[27] Mahdavi M , Fesanghary M , Damangir E. An improved harmony search algorithm for solving optimization problems. Appl Math Comput. 2007;188:1567‐1579.

[28] Brun R , Rademakers F. ROOT–An object oriented data analysis framework. Nucl Instrum Methods Phys Res, Sect A. 1997;389:81‐86.

[29] Antcheva I , Ballintijn M , Bellenot B , et al. ROOT ‐ A C++ framework for petabyte data storage, statistical analysis and visualization. Comput Phys Commun. 2009;180:2499‐2512.

[30] Carlino A , Stock M , Zagler N , et al. Characterization of PTW‐31015 PinPoint ionization chambers in photon and proton beams. Phys Med Biol. 2018;63:185020. 30152791 10.1088/1361-6560/aadd39

[31] Stock M , Georg D , Ableitinger A. The technological basis for adaptive ion beam therapy at MedAustron: Status and outlook. Zeitschrift für Medizinische Physik. 2018;28:196‐210. 29107440 10.1016/j.zemedi.2017.09.007

[32] Resch AF , Elia A , Fuchs H , et al. Evaluation of electromagnetic and nuclear scattering models in GATE/Geant4 for proton therapy. Med Phys. 2019;46:2444‐2456. 30870583 10.1002/mp.13472 PMC6850424

[33] Sommerer F , Parodi K , Ferrari A , Poljanc K , Enghardt W , Aiginger H. Investigating the accuracy of the FLUKA code for transport of therapeutic ion beams in matter. Phys Med Biol. 2006;51:4385‐4398. 16912388 10.1088/0031-9155/51/17/017

